# A multi-component, adaptive Working Memory Assessment Battery (WoMAB): validation and norms in an Italian population sample

**DOI:** 10.1007/s10072-021-05416-7

**Published:** 2021-06-29

**Authors:** Fabrizio Pasotti, Giulia De Luca, Edoardo Nicolò Aiello, Chiara Gramegna, Marco Di Gangi, Giuseppe Foderaro, Marcello Gallucci, Elena Biglia, Gabriella Bottini

**Affiliations:** 1 Department of Brain and Behavioural Sciences, University of Pavia, Pavia, Italy; 2 School of Medicine and Surgery, University of Milano-Bicocca, Monza, Italy; 3 PhD in Neuroscience, University of Milano-Bicocca, Monza, Italy; 4 Department of Psychology, University of Milano-Bicocca, Milano, Italy; 5 Studio Di Gangi & Vicini Psicologia Psicoterapia e Neuropsicologia, Minusio, Switzerland; 6 Neurocentro Della Svizzera Italiana, Ente Ospedaliero Cantonale Ospedale Regionale Di Lugano, Lugano, Switzerland; 7 Milan Center for Neuroscience (NeuroMI), Milano, Italy; 8 Cognitive Neuropsychology Centre, ASST Grande Ospedale Metropolitano Niguarda, Milan, Italy

**Keywords:** Working memory, Executive functioning, Normative data, Validation, Ecological validity, Neuropsychological assessment

## Abstract

**Background:**

Working memory (WM) abilities are frequently impaired in neurological disorders affecting fronto-parietal cortical/sub-cortical structures. WM deficits negatively influence interventional outcomes and everyday functioning. This study thus aimed at the following: (a) developing and standardizing an ecologically valid task for WM assessment ( Ice Cream Test, ICT); (b) validating and norming a novel WM test (Digit Ordering Test, DOT), as well as providing updated norms for digit span (DS) tasks, in an Italian population sample; (c) introducing a novel scoring procedure for measuring WM.

**Methods:**

One-hundred and sixty-eight Italian healthy participants—73 male, 95 females; age: 48.4 ± 19.1 (18–86); education: 12.1 ± 4.8 (4–21)—underwent a thorough WM assessment—DOT, ICT, and both forward and backward DS tasks (FDS, BDS). The ICT requires participants to act as waiters who have to keep track of customers’ orders. For each task, WM and total (T) outcomes were computed, i.e., the number of elements in the longest sequence and that of recalled sequences, respectively. Norms were derived via the equivalent score (ES) method.

**Results:**

DS ratios (DSRs) were computed for both WM/S and T outcomes on raw DS measures (BDS divided by FDS). Age and education significantly predicted all WM tasks; sex affected FDS and DSR-T scores (males > females). WM measures were highly internally related.

**Discussion:**

The present work provides Italian practitioners with a normatively updated, multi-component, adaptive battery for WM assessment (WoMAB) as well as with novel outcomes which capture different WM facets—WM capacity and attentive monitoring abilities.

## Introduction

Working memory (WM) comprises a set of high-order, non-instrumental limited-capacity cognitive functions that allow “temporary storage and manipulation of information necessary for […] complex cognitive tasks” [2].

The original multi-component model [3] identifies a central executive (CE) component and two modality-specific sub-components: the phonological loop (PL) and the visuospatial sketchpad (VSS)—processing verbal and visual information, respectively. The CE is a control system of limited capacity that supports complex cognitive activities by suppressing irrelevant information; it allocates attentive resources and allows alternating between different tasks. The PL is a modular subsystem that retains the memory online and prevents it from decaying through both vocal and sub-vocal rehearsals. The VSS temporarily stores and processes visual and spatial information. Most recent formulations [31] introduce a further component, i.e., the episodic buffer—a multi-modal limited-capacity system integrating information from the other components into a unitary episodic representation. WM functioning emerges from the interaction between perceptual and attentive mechanisms and representations stored in the long-term memory system [11, 12].

Converging evidence from neuroimaging and brain injury studies hint at a widespread bilateral fronto-parietal both cortical and sub-cortical network being the neural substrate of WM [18, 25, 27, 33].

WM deficits are thus often associated with focal or diffuse brain damages; hence, the neuropsychological assessment constitutes a relevant aspect for cognitive rehabilitation [13, 20, 28]. Several studies have found that WM deficits impair the activities of daily living and affect rehabilitation outcomes [14, 23].

In clinical practice, the digit span (DS) [36] is one of the most widely used tasks to measure the capacity of the auditory-verbal component in WM. According to Baddeley’s model [31], the forward (FDS, forward DS) version evaluates the short-term, passive retention of verbal stimuli, whereas the backward version (BDS, backward DS) requires maintaining and actively manipulating information in order to reproduce in reverse order the sequence presented. Both forward and backward versions of the digit span have been validated and normed in Italy [24, 26].

Another instrument for assessing auditory-verbal WM is the Digit Ordering Test (DOT) [10, 17, 37], which requires clients to listen to a series of randomly ordered digits and then to recall items in ascending order immediately after their presentation. No Italian standardizations of the DOT are available so far.

Evidence regarding the influence of WM deficits on daily functioning highlights the relevance of ecological validity in cognitive testing [5]. Available tests for the assessment of WM may fail to detect its dysfunction in everyday life [35].

We aimed at developing a composite WM assessment battery (WoMAB) and more specifically at the following: (a) standardizing a novel task that investigates auditory-verbal WM from an ecological perspective; (b) validating and norming the DOT in Italian healthy individuals; and (c) providing updated normative data for DS tasks. A novel scoring procedure will be also proposed: WM and total (T) outcomes, i.e., the number of elements in the longest sequence and that of recalled sequences, respectively. The underlying hypothesis is that WM scores reflect a measure of the auditory-verbal WM capacity, whereas T scores provide insight into attentive monitoring abilities during task execution.

## Methods

### Participants

One-hundred and seventy-three Italian native-speakers individuals were initially recruited from different regions of both Northern and Southern Italy, as well as from the Canton Ticino region of Switzerland. Sample stratification is displayed in Table 1.

**Table 1:** Sample stratification for age, education, and sex

Education		Age						
		35 ≤	36–45	46–55	56–65	66–75	76–85	≥ 86
5 ≤	M/F	0/0	0/0	0/1	3/1	4/8	4/6	0/0
6–11		2/2	5/1	4/13	7/5	4/3	0/1	1/0
12–16		9/11	3/4	2/6	4/1	1/1	0/1	0/0
≥ 17		11/21	1/0	3/3	4/6	1/0	0/0	0/0

Notes: Cells show male/female ratio for each co-occurrence

Inclusion criteria were as follows: (a) age between 20 and 90 years; (b) years of education between 5 and 18; (c) an adjusted scores on the Mini-Mental State Examination (MMSE) above the established cut-off [21, 22].

Participants were excluded if presenting with neurological disorders, traumatic brain injury, psychiatric disorders, previous brain surgeries, drug abuse, learning disabilities, psychotropic drug treatment, and visual/auditory impairments (participants with corrected-to-normal vision and audition were included).

After applying inclusion/exclusion criteria, *N* = 168 individuals were included.

Participants provided written informed consent before being enrolled. The study was approved by the Ethics Committee of the University of Pavia and conducted in accordance with the Declaration of Helsinki.

### Materials

Four auditory-verbal WM tests were administered whose order was counterbalanced across participants to avoid carry-over effects.

Each task started with warming-up trials. Mistakes on preliminary items could be corrected, although without providing execution strategies. Stimuli were pronounced at the rate of one per second, with neutral intonation. Participants were given 15 s to recall the items. Two lists of the same length were administered; the task was interrupted after two consecutive fails. No cues were provided but self-corrections were accepted. Recalled sequences containing intrusions were scored as 0. Both WM and T outcomes were computed for each WM task.

The Ice Cream Test (ICT) is a novel ecologically valid tool investigating auditory-verbal WM. Participants were required to act as if they were waiters in an ice cream shop who have to keep track of customers’ orders. Each customer will order a single ice cream flavor; it is required to tell, within 15 s, how many ice creams have to be prepared for each flavor. ICT-WM outcome ranges 0–10 (longest sequence) and ICT-T 0–16 (recalled sequences).

FDS and BDS tasks were adapted from Monaco et al. [24]. Additionally, the present version introduced one two-digit warming-up sequence. Contrarily to other WM outcomes, that for the FDS was named “Span” (FDS-S)—as not unequivocally targeting WM abilities. FDS-S ranges 0–9 (longest sequence) and FDS-T 0–16 (recalled sequences). BDS-WM ranges 0–8 (longest sequence) and BDS-T 0–14 (recalled sequences).

The DOT [10, 17, 37] consists of presenting a list of randomly ordered digits that have to be recalled in ascending order. DOT-WM outcome ranges 0–8 (longest sequence) and DOT-T 0–12 (recalled sequences).

Test protocols will be provided to interested practitioners upon request to the corresponding author.

### Statistical analyses

By conservatively assuming a small-to-medium size (*f*^2^ = 0.10) of background predictors effects (*df*numerator = 3) [6, 24, 26], the minimum sample size was set at *N* = 146 via a power analysis for multiple linear regression analyses [32] (R package *pwr*) [9]. α was set at 0.05 and 1-β at 0.9; *N* was yielded by *df*numerator + *df*denominator + 1.

Skewness and kurtosis statistics were regarded as suggestive of a violation of the assumption of normality if >|1| and |3|, respectively [19].

Associations of interest between quantitative variables were assessed by means of either Pearson’s or Spearman’s coefficients. Bonferroni correction for multiple comparisons was applied if adequate.

Norms were drawn by adopting the equivalent score (ES) method [8, 34], a regression-based approach adjusting raw scores (RSs) for significant predictors of interest (or their transforms) and then allotting adjusted scores (ASs) into a 5-level ability scale: ES = 0 ( “abnormal”); ES = 4 (“high-end normal”); ES = 1, 2, and 3 (respectively, “borderline”, “low-end normal”, “normal”). Outer and inner tolerance limits (oTL; iTL) were computed to provide an interval estimate for the cut-off (ASs < oTL fall within ES = 0). Average ESs (AES) [7] were computed for both T and WM/S outcomes in order to provide a global estimate of attentive monitoring and WM capacity across tasks.

R 3.6.3 [30] was used for implementing the analyses. Regression studies and calculations of both TLs and ES threshold were implemented as described in Aiello & Depaoli [1].

## Results

Participants’ background features and cognitive scores are summarized in Table 2.

**Table 2:** Participants’ background features and WM measures

	Background features	
* N*	168	
Sex (M/F)	73/95	
Age (years)	48.41 ± 19.08 (18–86)	
Education (years)	12.09 ± 4.8 (4–21)	
MMSE	28.44 ± 2.31 (17–30)	
WM measures	T	WM/S
DOT	7.33 ± 2.45(1–14)	6.25 ± 1.48 (2–12)
ICT	7.29 ± 2.81 (2–16)	6.32 ± 1.81 (3–11)
FDS	9.98 ± 2.48 (4–16)	6.45 ± 1.34 (4–9)
BDS	7.07 ± 2.73 (2–14)	5.02 ± 1.5 (2–8)
DSR	71 ± .21 (.22–1.27)	.78 ± .18 (.18–1.25)

Notes: *F*, female; *M*, male; *MMSE*, Mini-Mental State Examination; *DOT*, Digit Ordering Test; *ICT*, Ice Cream Test; *FDS*, forward digit span; *FDS*, backward digit span; *DSR*, digit span ratio; *T*, Total; *WM*, working memory; *S*, span

In agreement with Monaco et al.’s [24], the ratios between FDS and BDS tasks were computed (by dividing BDS measures by FDS ones) for both T al WM/S scores (DSR-T,DSR-WM/S).

Non-derived WM measures were highly internally related (0.39 ≤ *r*(168) ≤ 0.96; *p* < 0.001)—even when adjusting the significance threshold (α_adjsusted_ = 0.05/28 = 0.002).

Ratios were associated with all remaining WM outcomes (0.23 ≤ *r*(168) ≤ 0.72; 0.003 ≤ *p* ≤ 0.001), whereas not with FDS measures.

All WM/S and T measures were negatively associated with age (range: − 0.32 ≤ *r*(168) ≤  − 0.59; *p* < 0.001) whereas positively with education (range: 0.2 ≤ *r*(168) ≤ 0.46; *p* ≤ 0.011). Males outperformed females on the BDS-T, BDS-WM, and DSR-T (range: 2.17 ≤ *t*(166) ≤ 2.56; 0.011 ≤ *p* ≤ 0.035); no other sex differences were detected.

When simultaneously tested, age and education transforms revealed to be predictive of DOT (-T and -WM), ICT (-T and -WM), and FDS (-T and -S) scores (age: range: |.28|≤ *β* ≤|.37|; |3.03|≤ *t* ≤|4.22|; *p* ≤ 0.003; education: range: − 0.35 ≤ *β* ≤  − 0.24; − 3.97 ≤ *t* ≤  − 2.7; *p* ≤ 0.008). Within a multiple regression model, sex, age, and transformed education were found to be predictive of BDS-T (sex: *β* =  − 0.21; *t* =  − 3.48; *p* = 0.001; age: *β* =  − 0.47; *t* =  − 5.93; *p* < 0.001; education: *β* =  − 0.21; *t* =  − 2.71; *p* = 0.008) and BDS-WM (sex: *β* =  − 0.18; *t* =  − 2.87; *p* = 0.005; age: *β* =  − 0.46; *t* =  − 5.61; *p* < 0.001; education: *β* =  − 0.19; *t* =  − 2.38; *p* = 0.018). With respect to ratios, sex and transformed age were found to be predictive of DSR-T scores (sex: *β* =  − 0.08; *t* =  − 2.49; *p* = 0.014; age: *β* =  − 0.16; *t* =  − 4.6; *p* < 0.001), whereas only inverse age significantly predicted DSR-WM/S scores (*β* = 4.08; *t* = 3.39; *p* = 0.001).

The mean AES scores were 3.06 ± 0.7 (0.6–4) and 3.11 ± 0.7 (0.6–4) for WM and T outcomes, respectively. No association with either age or education was found with respect to both AESs. However, AES-T was significantly higher (*t*(166) = 2.4; *p* = 0.018) for males than for males.

Correction coefficients for selected co-occurrences of background predictors along with equations for adjusting RSs are reported in Table 3 (DOT and ICT), Table 4 (FDS and BDS), and Table 5 (DSR). Normative values for all measures are reported in Table 6. For AESs, only TLS are provided [7].

**Table 3:** Adjustment grids according to age and education for Digit Ordering Test (DOT) and Ice Cream Test (ICT) total (T) and working memory (WM) raw scores

	Education	Age										
		35	40	45	50	55	60	65	70	75	80	85
DOT												
T	5	1.08	1.21	1.35	1.5	1.67	1.86	2.07	2.31	2.6	2.96	3.42
	8	− .13	-	.14	.29	.46	.65	.86	1.11	1.4	1.75	2.21
	11	− .67	− .54	− .41	− .25	− .09	.1	.31	.56	.85	1.2	1.66
	13	− .90	− .77	− .63	− .48	− .31	− .12	.09	.33	.62	.98	1.44
	16	− 1.13	− 1	− .86	− .71	− .54	− .36	− .14	.1	.39	.75	1.2
	18	− 1.24	− 1.11	− .97	− .82	− .65	− .47	− .25	− .01	.28	.64	1.09
WM	5	.63	.68	.73	.8	.88	.98	1.1	1.23	1.39	1.57	1.78
	8	− .04	-	.06	.13	.21	.31	.42	.56	.72	.9	1.1
	11	− .34	− .3	− .25	− .18	− .1	−	.12	.26	.41	.59	.8
	13	− .47	− .43	− .37	− .31	− .22	− .12	− .01	.13	.29	.47	.67
	16	− .6	− .56	− .5	− .44	− .35	− .25	− .14	-	.16	.34	.54
	18	− .66	− .62	− .57	− .5	− .42	− .32	− .2	− .1	.1	.28	.48
ICT												
T	5	.91	1.08	1.26	1.46	1.68	1.92	2.2	2.52	2.9	3.37	3.97
	8	− .28	− .12	.07	.27	.49	.73	1.01	1.33	1.71	2.18	2.78
	11	− .82	− .66	− .48	− .28	− .06	.19	.47	.79	1.17	1.64	2.24
	13	− 1.05	− .88	− .7	− .5	− .28	− .03	.25	.57	.95	1.41	2.01
	16	− 1.28	− 1.11	− .93	− .73	− .51	− .26	.02	.34	.72	1.19	1.79
	18	− 1.39	− 1.22	− 1.04	− .84	− .62	− .37	− .09	.23	.61	1.07	1.68
WM	5	.48	.55	.63	.73	.85	1	1.18	1.38	1.62	1.89	2.2
	8	− .17	− .11	− .02	.08	.2	.35	.53	.73	.97	1.24	1.54
	11	− .47	− .4	− .32	− .22	− .09	.05	.23	.44	.67	.94	1.25
	13	− .59	− .52	− .44	− .34	− .22	− .07	.11	.31	.55	.82	1.13
	16	− .71	− .65	− .57	− .47	− .34	− .19	− .02	.19	.42	.7	1
	18	− .77	− .71	− .63	− .53	− .4	− .25	− .08	.13	.36	.63	.94

DOT-T adjusted score = raw score − 1.590625 * [ln(100 − age) − 3.862482] + 16.07802 * [(1/education) − .101788]. DOT-WM adjusted score **=** raw score + .000002* [(age^3) − 166,465.803571] + 8.9739 * [(1/education) − .101788]. ICT-T adjusted score = raw score − 2.087459 * [ln(100 − age) − 3.862482] + 15.88892 * [(1/education) − .101788]. ICT-WM adjusted score = raw score + .000003 * [(age^3) − 166,465.803571] + 8.691697*[(1/education) − .101788]. Significant decimals of adjustment factors are displayed. Adjustment factors have been extracted from the aforementioned formula and do not always reflect empirical co-occurrence

**Table 4:** Adjustment grids according to age and education for forward and backward digit span (F/BDS) total (T) and span/working memory (S/WM) raw scores

	Education		Age										
			35	40	45	50	55	60	65	70	75	80	85
FDS													
T		5	.6	.77	.95	1.15	1.37	1.61	1.89	2.21	2.59	3.05	3.65
		8	− .35	− .19	− .01	.19	.41	.66	.93	1.26	1.64	2.1	2.7
		11	− .79	− .62	− .44	− .24	− .02	.22	.5	.82	1.2	1.67	2.27
		13	− .97	− .8	− .62	− .42	− .2	.05	.32	.64	1.02	1.49	2.09
		16	− 1.15	− .98	− .8	− .6	− .38	− .14	.14	.46	.84	1.31	1.9
		18	− 1.24	− 1.07	− .89	− .69	− .47	− .23	.05	.37	.75	1.22	1.82
S		5	.23	.29	.37	.47	.6	.75	.92	1.13	1.37	1.64	1.94
		8	− .23	− .17	− .08	.02	.14	.29	.47	.67	.91	1.18	1.48
		11	− .44	− .37	− .29	− .19	− .07	.08	.26	.46	.7	.97	1.28
		13	− .52	− .46	− .38	− .28	− .15	-	.17	.38	.61	.88	1.19
		16	− .61	− .55	− .47	− .36	− .24	− .09	.08	.29	.53	.8	1.1
		18	− .65	− .59	− .51	− .41	− .28	− .13	.04	.25	.48	.75	1.06
BDS													
T	♂	5	− .39	− .06	.26	.59	.92	1.25	1.58	1.91	2.24	2.56	2.89
		8	− 1.21	− .89	− .56	− .23	.1	.43	.76	1.09	1.41	1.74	2.07
		11	− 1.59	− 1.26	− .93	− .6	− .27	.06	.38	.71	1.04	1.37	1.7
		13	− 1.74	− 1.41	− 1.08	− .76	− .43	− .1	.23	.56	.89	1.22	1.54
		16	− 1.9	− 1.57	− 1.24	− .91	− .58	− .26	.07	.4	.73	1.06	1.39
		18	− 1.97	− 1.65	− 1.32	− .99	− .66	− .33	-	.32	.65	.98	1.31
	♀	5	.78	1.11	1.44	1.77	2.1	2.42	2.75	3.08	3.41	3.74	4.07
		8	− .04	.29	.62	.95	1.27	1.6	1.93	2.26	2.59	2.92	3.24
		11	− .41	− .08	.24	.57	.9	1.23	1.56	1.89	2.21	2.54	2.87
		13	− .57	− .24	.09	.42	.75	1.08	1.4	1.73	2.06	2.39	2.72
		16	− .72	− .4	− .07	.26	.59	.92	1.25	1.58	1.9	2.23	2.56
		18	− .8	− .47	− .14	.19	.51	.84	1.17	1.5	1.83	2.16	2.48
WM	♂	5	− .21	− .04	.14	.32	.49	.67	.84	1.02	1.2	1.37	1.55
		8	− .62	− .45	− .27	− .1	.08	.26	.43	.61	.79	.96	1.14
		11	− .81	− .63	− .46	− .28	− .11	.07	.25	.42	.6	.78	.95
		13	− .89	− .71	− .53	− .36	− .18	− .01	.17	.35	.52	.7	.88
		16	− .97	− .79	− .61	− .44	− .26	− .08	.09	.27	.44	.62	.8
		18	− 1	− .83	− .65	− .48	− .3	− .12	.05	.23	.41	.58	.76
	♀	5	.34	.52	.7	.87	1.05	1.22	1.4	1.58	1.75	1.93	2.11
		8	− .07	.11	.29	.46	.64	.81	.99	1.17	1.34	1.52	1.7
		11	− .25	− .08	.1	.27	.45	.63	.8	.98	1.16	1.33	1.51
		13	− .33	− .15	.02	.2	.37	.55	.73	.9	1.08	1.26	1.43
		16	− .41	− .23	− .06	.12	.3	.47	.65	.82	1	1.18	1.35
		18	− .45	− .27	− .09	.08	.26	.43	.61	.79	.96	1.14	1.32

Notes: *M*, male; *F*, female; FDS-T-adjusted score = raw score − 2.082331*[ln(100 − age) − 3.862482] + 12.71517*[(1/education) − .101788]. FDS-S adjusted score** = **raw score + .000003*[(age^3) − 166,465.803571] + 6.09889*[(1/education) − .101788]. BDS-T adjusted score** = **raw score + .065689*(age − 48.41071) + 10.95221*[(1/education) − .101788] + .587136 if female; − .587136 if male. BDS-WM adjusted score** = **raw score + .035249*(age − 48.41071) + 5.473036*[(1/education) − .101788] + .278456 if female; − .278456 if male. Significant decimals of adjustment factors are displayed. Adjustment factors have been extracted from the aforementioned formula and do not always reflect empirical co-occurrences

**Table 5:** Adjustment grids according to age, education, and sex for the backward digit span (BDS) total (T) and Working Memory (WM) raw scores

DSR		Age										
		35	40	45	50	55	60	65	70	75	80	85
T	♂	− .074	− .053	− .035	− .018	− .003	.01	.023	.034	.045	.055	.065
	♀	-	.021	.04	.056	.071	.085	.097	.109	.12	.13	.139
WM/S		− .015	-	.011	.02	.027	.034	.039	.043	.047	.051	.054

Notes. DSR-T adjusted score** = **raw score + .156681*(ln(age) − 3.791483) − .037286 if M + .037286 if F. DSR-WM/S adjusted score** = **raw score − 4.081518*((1/age) − .024893). Significant decimals of adjustment factors are displayed. Adjustment factors have been extracted from the aforementioned formula and do not always reflect empirical co-occurrences

**Table 6:** Equivalent scores for DOT, ICT, FDS, BDS and RDS adjusted scores

	oTL	iTL	Equivalent scores				
			0	1	2	3	4
DOT-T	3.27	4.72	≤ 3.27	3.28–4.72	4.73–5.76	5.77–7.35	≥ 7.36
DOT-WM	4.05	4.5	≤ 4.05	4.064.57	4.58–5.42	5.43–6.31	≥ 6.32
ICT-T	2.48	3.7	≤ 2.48	2.49–4.14	4.15–5.7	5.71–7.03	≥ 7.04
ICT-WM	3.69	4.17	≤ 3.69	3.7–4.22	4.23–5.19	5.2–6.19	≥ 6.2
FDS-T	6.42	7.35	≤ 6.42	6.43–7.4	7.41–8.49	8.5–9.88	≥ 9.89
FDS-S	4.45	5.17	≤ 4.45	4.46–5.22	5.23–5.58	5.59–6.31	≥ 6.32
BDS-T	3.35	4.26	≤ 3.35	3.36–4.38	4.39–5.67	5.68–7.16	≥ 7.17
BDS-WM	2.77	3.41	≤ 2.77	2.78–3.45	3.46–4.26	4.27–4.88	≥ 4.89
DSR-T	.393	.486	≤ .393	.394–.502	.503–.638	.639–.735	≥ .736
DSR-WM/S	.461	.541	≤ .461	.462–.55	.551–.659	.66–.786	≥ .787
AES-T	1.4	2.2	≤ .1.4	-	-	-	-
AES-WM/S	1.2	2.2	≤ .1.2	-	-	-	-

T=total; WM=Working Memory; S=Span; DOT=Digit Ordering Test; ICT=Icre Cream Test; FDS=Forward Digit Span; BDS=Backward Digit Span; DSR=Digit Span Ratio; AES=average Equivalent Score; oTL=outer tolerance limit; iTL=inner tolerance limit.

## Discussion

This work provides Italian neuropsychologists with a novel standardized tool for the ecological assessment of auditory-verbal WM abilities (ICT), as well as with norms and validity evidence for the DOT. Both ICT and DOT measures proved to converge with widespread WM measures (FDS and BDS).

Moreover, updated normative data are provided for both the FDS and the DBS. Cut-offs here reported are substantially comparable to those found by Monaco et al. [24] (FDS: 2.78 vs. 2.65,BDS: 4.46 vs. 4.26). Inconsistently with previous normative studies [24, 26], as well as with contributions on sex differences in WM abilities [16], males scored higher than females on BDS and DSR measures. The present findings thus counterbalance those hinting at a prominent male–female difference being detectable on visuospatial but not in phonological WM tasks [29].

It is also worth noting that the DSR in the present study differs from that of Monaco et al. [24] since it was computed on raw rather than adjusted scores.

This work also introduced a novel scoring procedure that provides insights about different facets of phonological WM-WM capacity (WM/S) and attentive monitoring abilities during task execution (T) [4, 15].

AESs here reported further contribute to the adaptive nature of this composite battery. Indeed, the WoMAB allows an in-depth profiling of WM abilities by yielding both single-task-level (ESs) and global (AESs) standardized scores with respect to considered outcomes (T and WM/S). Although both AESs proved to be independent of age and education [7], practitioners should nonetheless exert caution when interpreting AES-T measures due to sex differences.

A limitation has to be finally acknowledged regarding sample stratification: certain co-occurrences of age and education levels indeed happened to be poorly represented (e.g., highly educated individuals aged ≥ 75 years)—possibly due to sampling biases. This should lead to exercising attention when adjusting RSs of individuals with these background features. However, it is believed that the soundness of regression analyses as far as statistical power is concerned allows sufficiently adequate predictions of adjustment factors for the aforementioned co-occurrences too.

In conclusion, this study validated and normed the WoMAB, a multi-component, flexible battery for WM assessment in adult neurological populations. Its novel scoring procedure allows assessing both WM capacity (longest sequence) and task-related attentive processes (number of recalled sequences). Moreover, the WoMAB encompasses ecologically valid measures that can help practitioners evaluate the impact of WM deficits in patients’ daily activities.
